# The complete plastome sequence of the endangered orchid *Cymbidium macrorhizon* (Orchidaceae)

**DOI:** 10.1080/23802359.2017.1390411

**Published:** 2017-10-18

**Authors:** Young-Kee Kim, Myoung Hai Kwak, Myong Gi Chung, Hoe-Won Kim, Sangjin Jo, Jung-Yeon Sohn, Se-Hwan Cheon, Ki-Joong Kim

**Affiliations:** a Division of Life Sciences, Korea University, Seoul, Korea;; b Department of Plant Resources, National Institute of Biological Resources, Incheon, Korea;; c Division of Life Science and the Research Institute of Natural Science, Gyeongsang National University, Jinju, Korea

**Keywords:** Plastome, *Cymbidium macrorhizon*, orchidaceae, hemisaprophyte, endangered species

## Abstract

In this study, we determined the complete chloroplast sequence of *Cymbidium macrorhizon* Lindl. (Orchidaceae) (NCBI acc. no. KY354040), an endangered plant species protected by the national law of Korea. The gene order and number in the *C. macrorhizon* plastome were similar to a typical Orchid plastome. The complete plastome was 149,859 bp in length and consisted of a large single copy region of 85,187 bp and a small single copy region of 13,766 bp; these were separated by two inverted repeats of 25,453 bp. The plastome contained 103 genes of which 69 were protein-coding genes, 30 were tRNA genes and four were rRNA genes. Fourteen genes contained one intron and two genes (*clp*P, and *ycf*3) had two introns. The AT content of the *C. macrorhizon* plastome was 60.0% and a total of 62 simple sequence repeat regions were identified in the plastome. Phylogenetic analysis also identified *C. lancifolium* as a closely related sister to *C. macrorhizon*, suggesting that the hemisaprophytic nature of *C. macrorhizon* is derived recently from a common leafy ancestor.

*Cymbidium macrorhizon* Lindl., a terrestrial orchid in the genus *Cymbidium*, is native to tropical and subtropical Asia and northern Australia (Lee 2011). In nature, *C. macrorhizon* is very rare and has been designated as an endangered and protected species in Korea. The plants are less than 30 cm tall and, as they do not have any leaves, have been described as a hemisaprophytic orchid species (Yukawa and Stern 2002). The genus *Cymbidium* belongs to the subfamily Epidendroideae of the family Orchidaceae (Chase et al. 2016). To develop genetic markers for endangered *C. macrorhizon*, we obtained and analyzed its plastome sequence.

*Cymbidium macrorhizon* plant material was collected from its natural habitat of Wando-gun, Jeollanam-do in Korea under a collection permit from the environmental protection authority of the Korea Government. As this is a nationally endangered species, we were permitted to collect only one individual. Therefore, a representative specimen was not deposited in herbarium. But, extracted DNAs were deposited in the Plant DNA Bank in Korea (PDBK 2016-1500). A whole plant was ground into powder in liquid nitrogen and total DNA was extracted using a G-spin™II Plant Genomic DNA extraction kit (iNtRON, Seongnam, Korea). The complete plastome sequence was generated using Illumina MiSeq (San Diego, CA) and assembled after trimming by Geneious 6.1.8 (Kearse et al. 2012). Average sequence coverage of 248 times the plastome size was obtained. Annotations were performed using the National Center for Biotechnology Information (NCBI) BLAST and tRNAscan-SE programmes (Lowe and Eddy 1997). The complete plastome sequence was submitted to the NCBI database under the accession number KY354040.

The gene order and number in the *C. macrorhizon* plastome were similar to a typical angiosperm (Shinozaki et al. 1986; Kim and Lee 2004; Yi and Kim 2012) with the exception of the *ndh* gene family. A typical plant plastome contains 11 *ndh* genes, the *C. macrorhizon* plastome, however, contained only a single gene, *ndh*C. Ten *ndh* gene losses are not unique to *C. macrorhizon* and the losses occur commonly in other Orchidaceae plastomes (Chang et al. 2006; Wu et al. 2010; Lin et al. 2015). Therefore, the *C. macrorhizon* plastome contained 103 unique genes including 69 protein-coding genes, 30 tRNA genes and four rRNA genes. Fourteen genes had a single intron while the *clp*P and *ycf*3 genes had two introns. The length of the complete plastome of *C. macrorhizon* was 149,859 bp; this was composed of a large single copy region of 85,187 bp, a small single copy region of 13,766 of bp, and two inverted repeats of 25,453 bp. The *C. macrorhizon* plastome was approximately 10 kb shorter than a typical angiosperm plastome because of the losses of *ndh* gene family. The average AT content of the plastome was 60.0%. We identified a total of 62 simple sequence repeat (SSR) loci including 48 mono-SSRs, 11 di-SSRs and three tri-SSRs spread throughout the plastome.

Phylogenetic analyses were performed on a dataset that included 78 protein-coding genes (excluding *ycf*1) and four rRNA genes extracted from 46 taxa in the NCBI database and *C. macrorhizon*. The gaps for lost genes were treated as missing bases. The 82 gene sequences were aligned with MUSCLE in Geneious 6.1.8; the aligned data matrix consisted of a total of 70,692 bp. This alignment was used for phylogenetic analysis using RAxML version 7.7.1 (Stamatakis et al. 2008). An ML tree was obtained with an ML estimation value of −307032.696860. The sister group relationship between *C. macrorhizon* and *C. lancifolium* was supported by 100% bootstrap value (Figure 1). The *C. macrorhizon*–*C. lancifolium* clade nested firmly within the *Cymbidium* group. While *C. macrorhizon* has been described as a saprophytic orchid species (Yukawa and Stern 2002), our plastome data showed an almost complete plastome gene content. The sister group relationship between the saprophytic *C. macrorhizon* and the leafy *C. lancifolium*, identified in our phylogenetic tree, suggest that the saprophytic nature are recently derived in the *C. macrorhizon* lineage (Figure 1). This tree also identified *Oncidium sphacelatum* and *Erycina pusilla* as sister genera to the monophyletic *Cymbidium* genus.

**Figure 1.**
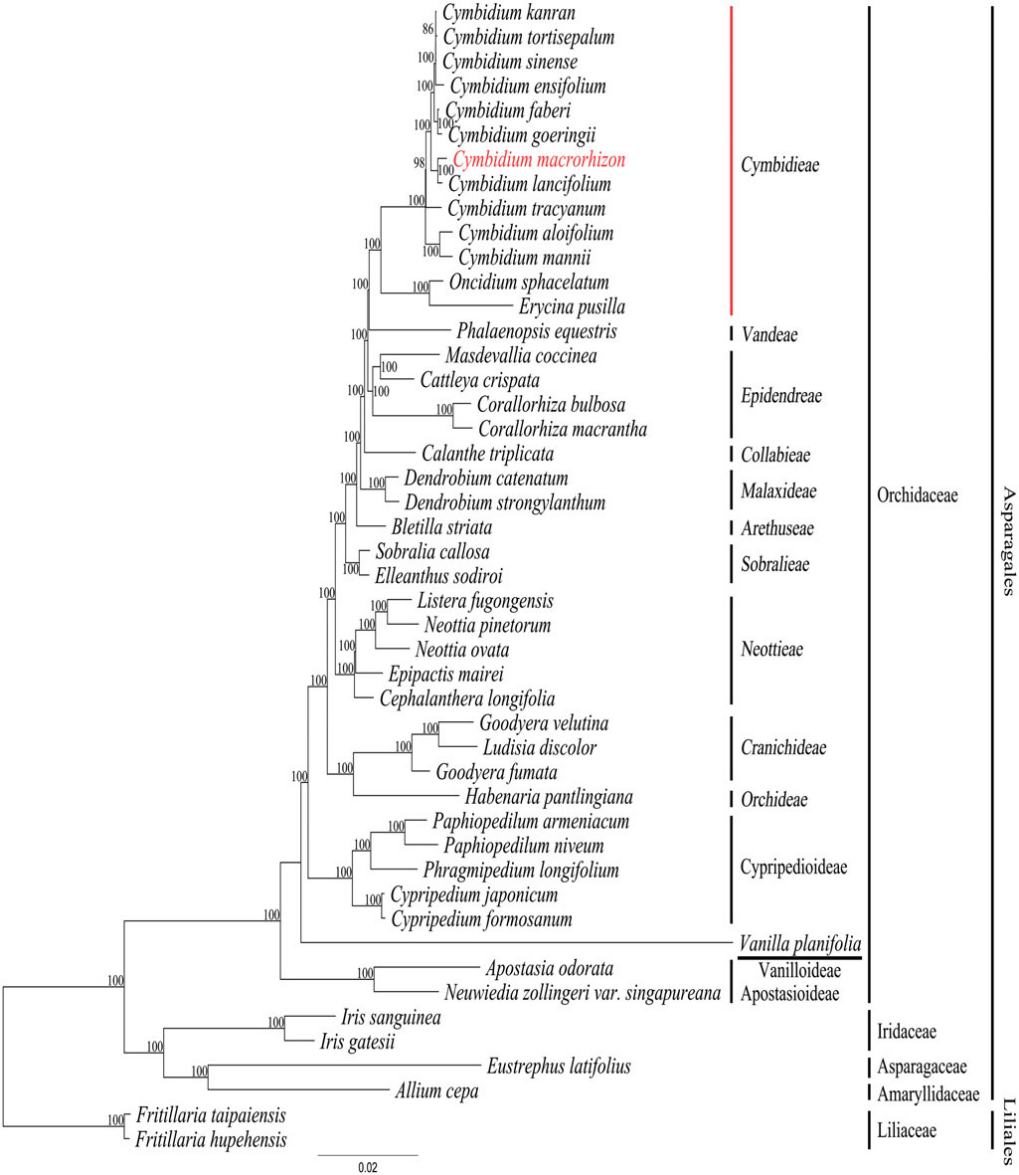
Chlroplast phylogenetic tree of Asparagales. A maximum likelihood tree (ML) inferred from analysis of alignment data containing 78 protein coding genes and four rRNA genes in 47 plastome sequences. The number above or below or each node indicate bootstrap value. Genbank accession numbers of taxa are shown below, *Allium cepa* (NC024813), *Apostasia odorata* (NC030722), *Bletilla striata* (NC028422), *Calanthe triplicata* (NC024544), *Cattleya crispata* (NC026568), *Cephalanthera longifolia* (NC030704), *Corallorhiza bulbosa* (NC025659), *C. macrantha* (NC025660), *C. aloifolium* (NC021429), *C. ensifolium* (NC028525), *C. faberi* (NC027743), *C. goeringii* (NC028524), *C. kanran* (NC029711), *C. lancifolium* (NC029712), *C. manii* (NC021433), *C. macrorhizon* (KY354040), *C. sinense* (NC021430), *C. tortisepalum* (NC021431), *C. tracyanum* (NC021432), *Cypripedium formosanum* (NC026772), *C. japonicum* (NC027227), *Dendrobium catenatum* (NC024019), *D. strongylanthum* (NC027691), *Elleanthus sodiroi* (NC027266), *Epipactis mairei* (NC030705), *Erycina pusilla* (NC018114), *Eustrephus latifolius* (NC025305), *Fritillaria hupehensis* (NC024736), *F. taipaiensis* (NC023247), *Goodyera fumata* (NC026773), *G. velutina* (NC029365), *Habenaria pantlingiana* (NC026775), *Iris gatesii* (NC024936), *I. sanguinea* (NC029227), *Listera fugongensis* (NC030711), *Ludisia discolor* (NC030540), *Masdevallia coccinea* (NC026541), *Neottia ovata* (NC030712), *N. pinetorum* (NC030710), *Neuwiedia zollingeri* var. *singapureana* (KM244735), *Oncidium sphacelatum* (NC028148), *Paphiopedilum armeniacum* (NC026779), *P. niveum* (NC026776), *Phalaenopsis equestris* (NC017609), *Phragmipedium longifolium* (NC028149), *Sobralia callosa* (NC028147), *Vanilla planifolia* (NC026778).
